# A chicken bioreactor for efficient production of functional cytokines

**DOI:** 10.1186/s12896-018-0495-1

**Published:** 2018-12-29

**Authors:** Lissa R. Herron, Clare Pridans, Matthew L. Turnbull, Nikki Smith, Simon Lillico, Adrian Sherman, Hazel J. Gilhooley, Martin Wear, Dominic Kurian, Grigorios Papadakos, Paul Digard, David A. Hume, Andrew C. Gill, Helen M. Sang

**Affiliations:** 1 The Roslin Institute, University of Edinburgh, Easter Bush, Midlothian, EH25 9RG UK; 2 Centre for Inflammation Research at the University of Edinburgh, The Queen’s Medical Research Institute, Edinburgh, EH16 4TJ UK; 3 Medical Research Council University of Glasgow Centre for Virus Research (CVR), University of Glasgow, Glasgow, G61 1QH UK; 4 Edinburgh Protein Production Facility, Wellcome Trust Centre for Cell Biology (WTCCB), University of Edinburgh, Edinburgh, EH9 3JR UK; 5 Mater Research Institute-University of Queensland, Translational Research Institute, Woolloongabba, QLD 4102 Australia; 6 School of Chemistry, Joseph Banks Laboratories, University of Lincoln, Lincoln, Lincolnshire LN6 7DL UK; 7 Roslin Technologies Limited, Roslin Innovation Centre, Easter Bush Campus, Midlothian, EH25 9RG UK

**Keywords:** Transgenic animals, Biologics, Recombinant proteins, Cytokines, Animal biotechnology

## Abstract

**Background:**

The global market for protein drugs has the highest compound annual growth rate of any pharmaceutical class but their availability, especially outside of the US market, is compromised by the high cost of manufacture and validation compared to traditional chemical drugs. Improvements in transgenic technologies allow valuable proteins to be produced by genetically-modified animals; several therapeutic proteins from such animal bioreactors are already on the market after successful clinical trials and regulatory approval. Chickens have lagged behind mammals in bioreactor development, despite a number of potential advantages, due to the historic difficulty in producing transgenic birds, but the production of therapeutic proteins in egg white of transgenic chickens would substantially lower costs across the entire production cycle compared to traditional cell culture-based production systems. This could lead to more affordable treatments and wider markets, including in developing countries and for animal health applications.

**Results:**

Here we report the efficient generation of new transgenic chicken lines to optimize protein production in eggs. As proof-of-concept, we describe the expression, purification and functional characterization of three pharmaceutical proteins, the human cytokine interferon α2a and two species-specific Fc fusions of the cytokine CSF1.

**Conclusion:**

Our work optimizes and validates a transgenic chicken system for the cost-effective production of pure, high quality, biologically active protein for therapeutics and other applications.

**Electronic supplementary material:**

The online version of this article (10.1186/s12896-018-0495-1) contains supplementary material, which is available to authorized users.

## Background

Since the development of recombinant human insulin, protein-based therapeutics have been the fastest-growing segment of the pharmaceutical industry. Three of the five highest revenue drugs in oncology, Rituxan, Avastin and Herceptin, are monoclonal antibodies [1]. The appearance of hundreds of non-antibody therapeutic proteins, including enzymes, cytokines and growth factors and blood proteins, validates predictions from 2004 [2] that this would be an area of significant growth. Between 2011 and 2016, 62 new therapeutic protein drugs were approved by the US FDA [3]. A further 27 were approved between September 2016 and December 2017 [4]. Although the unique benefits of biopharmaceuticals are clear, the costs to the health care system are becoming prohibitive [5]. Prices charged have traditionally been based upon patient benefit, but the cost of goods is also significant, especially in the transition to generics and biosimilars. Many biopharmaceuticals are produced using bacterial, yeast or mammalian cell culture expression systems. Current mammalian cell-culture based systems are increasingly optimised and industrialized [6]. Each system is constrained in some measure by yield, cost, complex purification procedures and appropriate post-translational modification when required for biological function.

Development of genetic modification systems for livestock offered the possibility of harnessing natural high-volume protein production systems (e.g. milk from farmed livestock and eggs from poultry) for the production of biologics. Transgenic sheep, goats and cows were generated that produced biopharmaceuticals in their milk, with the aim of establishing animal bioreactors for large scale production of high quality proteins, potentially at lower capital and production costs than cell culture systems [7, 8]. The first drug produced in a transgenic animal, antithrombin III in goat milk, was approved for use by the European Medicines Agency in 2006 and the FDA in 2009 [9]. However, there are limitations to the use of large mammals as bioreactors. They have few offspring, long gestation times and require a large amount of space and feed, all of which can make industrial scale-up difficult and expensive. It can also be difficult to purify a recombinant protein from milk due to the high content of lipids and other proteins [10]. Reabsorption of the protein by the animal can have deleterious effects, particularly if the human protein is biologically active in the host [11].

Interest in developing the laying hen as a bioreactor is based on the high protein synthetic capacity of the hen: an average 60 g egg contains nearly 3.5 g of protein in the egg white [12] and each hen may lay over 300 eggs per year. Chickens are relatively inexpensive to keep [10] and large flocks can be built quickly from small numbers of founder birds. In addition, chickens have closely-related glycosylation patterns to humans [12], which may result in reduced immunogenicity of biologics from egg white, as well as improving functionality of proteins where glycosylation is required for activity [13–15]. We showed previously that lentiviruses can be used for efficient production of transgenic chickens [16]. Transgene expression can be restricted to the oviduct (and thus egg white) using sequences from the ovalbumin gene, encoding the most abundant egg white protein [17]. While this approach allowed successful production of two biologically active proteins in egg white, the expression levels achieved were well below the > 1 g/L likely to be required as the basis of a commercially viable system. In addition, purification of recombinant proteins from egg white, a matrix characterized by high levels of other proteins including albumin and the gel-like glycoconjugate ovomucin, remained to be tackled [17]. Here we demonstrate the improved and optimized production of two new proteins, including a cytokine dimer, and robust purification protocols. Interferon α2a is a legacy, off-patent biologic used to treat certain forms of cancer and hepatitis [18–20] and was selected as a model for biosimilar production in the chicken system. Macrophage colony-stimulating factor (CSF1) is a candidate therapeutic protein in many areas of regenerative medicine [21]. Like interferon, CSF1 is a 4-helix bundle protein, structurally-related to many cytokines, including insulin, somatotrophin, placental lactogen, G-CSF, GM-CSF and erythropoietin (EPO) [22] that are in widespread clinical use. Immunoglobulin Fc fusion proteins are an emerging class of biopharmaceuticals [23, 24]. Several therapeutic 4 helix bundle proteins have been tested as fusion proteins with the Fc region to improve their circulating half-life and biological activity [24–28]. We recently produced a Fc fusion protein of CSF1 and demonstrated increased in vivo efficacy in rodent and large animal models, and potential as therapeutic in liver regeneration [29–32]. An additional attraction of the Fc tag in generation of therapeutic proteins in bioreactors is that it provides a generic tag for affinity purification. Here we demonstrate the optimal production, downstream processing and biological activity of recombinant proteins produced in the transgenic avian system. The chicken bioreactor clearly has the potential to address the cost and availability of therapeutic proteins for both human and veterinary medicine.

## Results

### Production of a high-purity, biologically active human cytokine

**Fig. 1:**
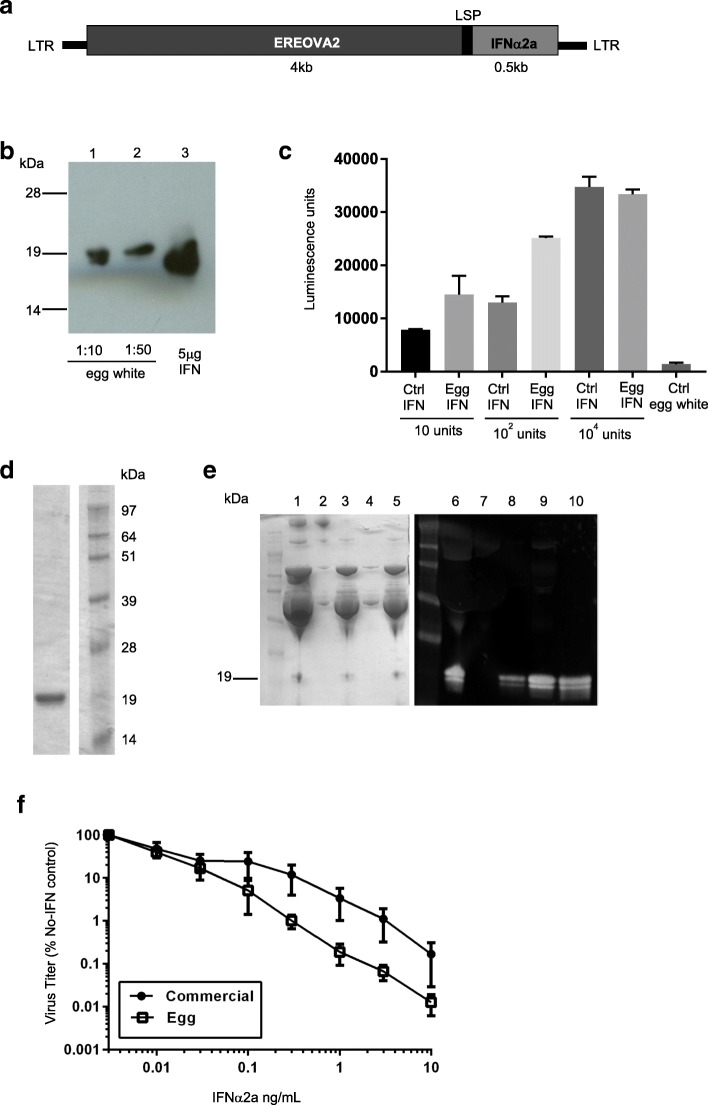
Expression of biologically-active human interferon α2a by transgenic hens **a** Schematic representation of the interferon α2a transgene; ovalbumin regulatory elements including the estrogen response element (ERE) and the steroid-dependent regulatory element (SDRE); lysozyme signal peptide (LSP) for secretion; the coding sequence of human interferon α2a; EIAV long terminal repeats (LTR). **b** Western blot of dilutions of transgenic egg white protein (lanes 1 and 2) and 5 μg of commercially-available interferon α2a protein (lane 3), stained with antibody for human interferon α2a. **c** Activity of whole egg white assayed for activation of the interferon-stimulated response element (ISRE) by measurement of luciferase. Egg white from transgenic hens was compared with egg white with a range of concentrations of control protein added, and wild type egg white as a negative control. Measurements were taken in triplicate. Graph shows mean + SEM. **d** Interferon α2a was purified from egg white and confirmed by reducing SDS-PAGE Instant Blue staining. **e** SDS-PAGE of 5 μL samples of egg white before ovomucin precipitation (lane 1), pellet after first centrifugation (lane 2), supernatant after first centrifugation (lane 3), pellet after pH adjustment and second centrifugation (lane 4), and supernatant after pH adjustment and second centrifugation (lane 5); and western blot of 10 μL samples from each stage of purification: Blue Sepharose load (lane 6), Blue Sepharose flow-through (lane 7), Blue Sepharose wash (lane 8), Blue Sepharose eluate (lane 9) and final pure protein (lane 10). **f** Purified interferon α2a from egg white and a purchased control protein were tested in a viral dose-inhibition assay against influenza A virus. Measurements were taken in triplicate. Graph shows mean ± SEM

Human interferon α2a is a cytokine commonly used for treatment of hepatitis and various cancers [18–20]. The 0.5 kb coding sequence for interferon α2a was synthesized and cloned into a replication-defective equine infectious anemia virus (EIAV) lentiviral vector with a modified form of the EREOVA promoter previously published [17]. To increase the level of expression, we reintroduced an additional 0.9 kb of the ovalbumin promoter sequence between the estrogen response element (ERE) and the steroid-dependent regulatory element (SDRE) (EREOVA2; Fig. 1a, Additional file 1: Figure S1). Transgenic birds were generated using this lentiviral vector with our established methods [17]. A single G1 male was identified that carried an intact transgene and a line established from this bird. The presence of interferon α2a in egg white in hens from this line was confirmed by western blot analysis with an antibody against human interferon α (Fig. 1b), and concentration measured by a commercial ELISA kit. Biological activity before purification was confirmed by applying diluted whole egg white to cells transfected with a reporter of interferon-stimulated response element (ISRE) driving luciferase expression (Fig. 1c).

A multi-step purification protocol was developed that involves clarification of ovomucin by pH reduction and its removal by centrifugation, and purification of the interferon α2a by HiTrap Blue capture followed by size exclusion chromatography (SEC) polishing; purity was confirmed by SDS-PAGE (Fig. 1d). Recovery of interferon α2a was approximately 15 mg from 100 mL of egg white, which is 60% of the original 25 mg/100 mL estimate determined by ELISA, with most of the loss occurring in the wash of the capture step; there is no observable protein loss in the ovomucin precipitation step or polishing step (Fig. 1e). Purity of > 95% was determined by reversed phase chromatography and SEC-multi-angle light scattering analyzes (Additional file 2: Figure S2).

The anti-viral properties of the purified interferon α2a were examined using a dose-inhibition assay against an H1N1 influenza A virus (A/Puerto Rico/8/1934 PR8) in A549 cells pre-treated with interferon α2a. The assay was performed as described previously [33] with commercially-available interferon α2a (produced in *E. coli*) as a control. The egg white interferon showed improved activity compared to the commercial protein (Fig. 1f), calculated to be as high as 1 × 10^9^ U/mg, an order of magnitude greater than the bacterially-produced protein.

### Simple purification of high quality, biologically active porcine growth factor-Fc fusion protein

**Fig. 2:**
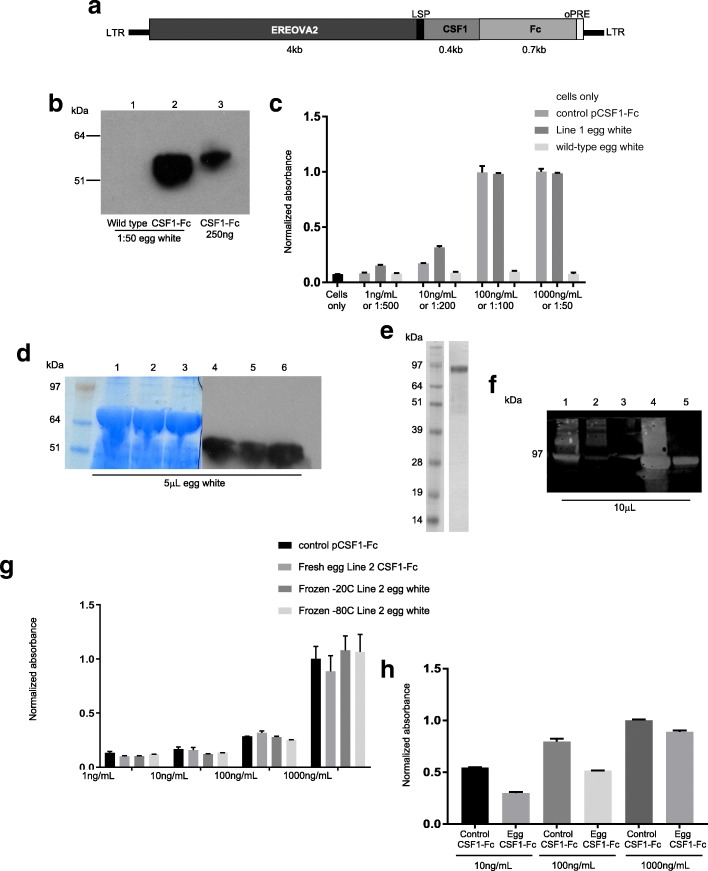
Expression of biologically-active porcine CSF1-Fc by transgenic hens. **a** Schematic representation of the pCSF1-Fc transgene, with HIV LTRs; a modified ovalbumin promoter containing the ERE and SDRE; LSP for secretion; the coding sequence of porcine CSF1; the coding sequence of porcine Fc; oPRE. **b** Egg white from wild type and transgenic hens were homogenized and diluted 1:50 in PBS (lanes 1 and 2) and separated under reducing conditions by SDS-PAGE with CHO-cell produced pure protein (lane 3) and transferred to nitrocellulose membrane and stained with anti-porcine Fc to confirm expression. **c** Activity of whole egg white was tested with a MTT cell survival assay. Whole egg white from transgenic hens was compared with wild type egg white with four concentrations of control protein added, while wild type egg white was used as a negative control. Measurements were taken in triplicate. Graph shows mean + SEM. **d** SDS-PAGE and western blot of 5 μL samples of egg white before ovomucin precipitation (lanes 1 and 4), supernatant after first centrifugation (lanes 2 and 5), and supernatant after pH adjustment and second centrifugation (lanes 3 and 6). **e** pCSF1-Fc dimer was purified from egg white as confirmed by non-reducing SDS-PAGE stained with Instant Blue. **f** Western blot of 10 μL samples from each stage of purification: MabSelect SuRe load (lane 1), MabSelect SuRe flow-through (lane 2), MabSelect SuRe wash (lane 3), MabSelect SuRe eluate (lane 4) and final pure protein (lane 5). **g** Activity of purified pCSF1-Fc from fresh egg white, egg white that had been frozen at -20 °C and -80 °C for at least 1 month before purification, and CHO-cell produced pCSF1-Fc was tested in a MTT Ba/F3-CSF1R cell-survival assay. Measurements were taken in triplicate. Graph shows mean + SEM. **h** Bone marrow from pigs was cultured in CHO-cell-produced pCSF1-Fc, pCSF1-Fc or without growth factor (cells only) for 7 days. MTT was used to assess cell viability. Measurements were taken in triplicate. Graph shows mean + SEM

Colony stimulating factor 1 (CSF1) is required for differentiation, proliferation and function of macrophages, and has shown promise as a therapeutic protein for a variety of indications, particularly in regenerative medicine [21, 29, 30]. Porcine CSF1 is active across mammalian species [34], and an Fc fusion protein of CSF1 was shown to have increased half-life and efficacy in vivo [30]. The coding sequence for porcine CSF1 fused to an antibody Fc region (pCSF1-Fc) [30] was synthesized and cloned into a pLenti6 (Invitrogen) lentiviral vector derived from human immunodeficiency virus (HIV), downstream of the EREOVA2 promoter and upstream of optimized woodchuck hepatitis virus posttranscriptional regulatory element [35] (oPRE) (Fig. 2a). Founder (G_0_) cockerels were generated [17] and bred to identify G_1_ transgenic birds. The presence of a single insertion of the intact transgene was confirmed in two hens and one cockerel by Southern blot analysis (Additional file 3: Figure S3). The two hens had the same insertion site (Line 1), while the cockerel had a different insertion (Line 2); all three were bred with stock birds to generate G_2_ hens. Expression of pCSF1-Fc in egg white was confirmed by SDS-PAGE and western blot with an antibody against porcine Fc (Fig. 2b), and expression was equivalent between two generations (Additional file 4: Figure S4). To test the functionality of pCSF1-Fc egg white, porcine CSF1 receptor was expressed in factor-dependent Ba/F3 cells [34] as previously described [30], making them dependent on functional CSF1-Fc for survival. Whole egg white from both Line 1 hens promoted survival and proliferation at similar concentrations to control pCSF1-Fc produced in CHO cells [30], while wild type egg white alone was unable to promote survival (Fig. 2c).

Egg white from pCSF1-Fc eggs was clarified of ovomucin as described for interferon purification. All of the protein came through in the supernatants (Fig. 2d). The active dimer form of pCSF1-Fc was purified by MabSelect SuRe capture followed by size exclusion chromatography (SEC) polishing, and purity confirmed by SDS-PAGE (Fig. 2e). Due to the difficulty in quantifying pCSF1-Fc in whole egg white by western blot and the lack of an ELISA test, concentration of protein was calculated based on the final recovery and estimates of protein loss through the purification process based on western blots of samples collected throughout the process (Fig. 2f). Purity was > 97%, as determined by reversed phase chromatography and SEC-multi-angle light scattering. The molecular mass average across the elution profile is 95.1 kDa with good protein mono-dispersity (Mw/Mn = 1.011) and no significant aggregation. Co-polymer model analysis indicated that peak mass was comprised of 85.6 kDa of protein and 9.5 kDa of oligosaccharide (~ 10% glycosylation). (Additional file 5: Figure S5). Mass spectrometry analysis confirmed the identity of pCSF1-Fc; presence of contaminating egg white proteins was below the limit of detection (Additional file 6: Table S1). The Ba/F3 cell survival assay was repeated with pCSF1-Fc purified from fresh egg white, egg white that had been stored at -20 °C for at least 1 month, and egg white that had been stored at -80 °C for at least 1 month. The control pCSF1-Fc and all purified egg pCSF1-Fc samples were able to promote cell survival at comparable levels (Fig. 2g), demonstrating that egg white can be collected and frozen for a significant period of time prior to purification without loss of recovery or activity. The ability of the purified protein to interact with the native CSF1 receptor was confirmed using mouse bone marrow cells. Both control and egg-derived pCSF1-Fc promoted cell differentiation at comparable levels (Fig. 2h).

**Fig. 3:**
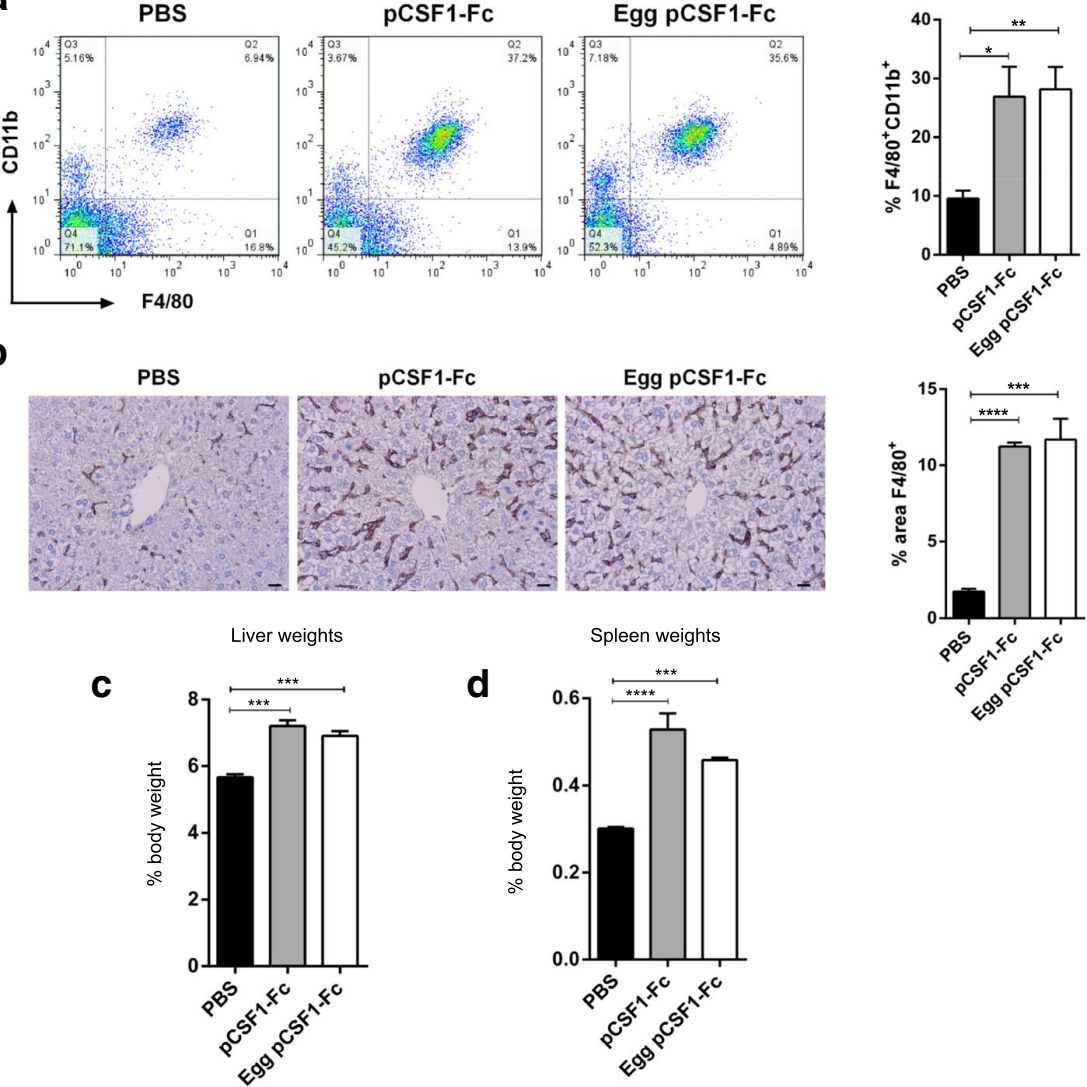
The effect of egg purified pCSF1-Fc treatment in mice is identical to that of pCSF1-Fc from CHO cells. Mice were injected with PBS, 1 μg/g pCSF1-Fc or 1 μg/g egg purified pCSF1-Fc for 4 days prior to sacrifice on day 5. **a** EDTA-blood was collected via cardiac bleeds and the percentage of F4/80^+^CD11b^+^ myeloid cells were determined by flow cytometry. Graph shows the mean + SEM. Significance is indicated by *p = 0.0163 and **p = 0.0037 using a t-test; *n* = 4. **b** Formalin-fixed paraffin-embedded livers were stained with an antibody against F4/80. The percentage of F4/80 staining was determined using ImageJ. Graph shows the mean + SEM. Significance is indicated by ***p = 0.0004 and ****p < 0.0001 using a test-test; n = 4. **c** Livers were weighed and percent body weight calculated. Graphs show the mean + SEM. Significance is indicated by ***p = 0.0004 using a t-test. There was no significant difference between the two pCSF1-Fc populations (*p* = 0.2482). **d** Spleens were weighed and percent body weight calculated. Graphs show the mean + SEM. Significance is indicated by ****p < 0.0001 and ***p = 0.0009 using a t-test. There was no significant difference between the two pCSF1-Fc populations (*p* = 0.1122)

To determine the activity of egg-derived pCSF1-Fc in vivo, mice were treated with 1 μg/g of either control or purified egg pCSF1-Fc, following which the livers, spleens and blood were analyzed for weight and histology as previously described [30]. Consistent with previous reports, both forms of pCSF1-Fc caused an increase in F4/80^+^CD11b^+^ cells in the blood (Fig. 3a) and F4/80^+^ tissue macrophages in the liver (Fig. 3b). There were also significant increases in the weights of the liver and spleen (Fig. 3c-d).

### Validation of the Fc-fusion approach by production of a potential human therapeutic cytokine

**Fig. 4:**
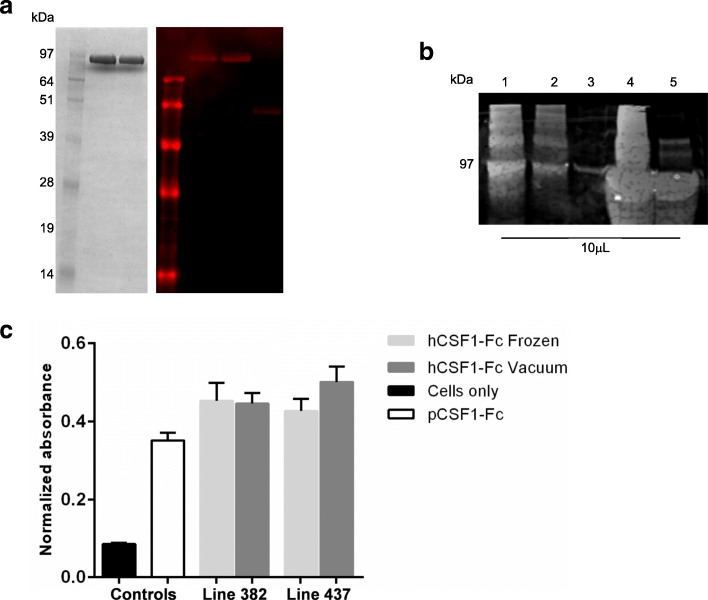
Expression of biologically-active human CSF1-Fc by transgenic hens. **a** Purity of 10 μL samples from 15 mL size exclusion fractions of hCSF1-Fc from egg white demonstrated by non-reducing SDS-PAGE stained with Instant Blue. Identity was confirmed by western blot transfer and staining with mouse anti-human CSF1 antibody and LICOR IRDye 680RD Donkey anti-Mouse IgG (H + L) secondary antibody. **b** Western blot of 10 μL samples from each stage of purification: MabSelect SuRe load (lane 1), MabSelect SuRe flow-through (lane 2), MabSelect SuRe wash (lane 3), MabSelect SuRe eluate (lane 4) and final pure protein (lane 5). **c** Bone marrow from pigs was cultured in purified pCSF1-Fc from egg white, purified hCSF1-Fc from egg white, and the same protein after vacuum drying and reconstitution, or without growth factor (cells only) for 7 days. MTT was used to assess cell viability. Measurements were taken in triplicate. Graph shows mean + SEM

Although pCSF1-Fc is active on human cells and might have short-term therapeutic applications in human patients and in veterinary medicine, it is likely to be immunogenic. Indeed, we have immunized mice with the CSF1-Fc protein to produce monoclonal antibodies that cross-react with human CSF1 (hCSF1; L.Waddell, DAH, manuscript in preparation). As a prelude to testing human clinical applications, we have generated additional chicken lines expressing a human CSF1-Fc (hCSF1-Fc) protein using the same vector system and employing the optimized purification protocol. The presence of a single insertion of the intact transgene was confirmed in two hens by Southern blot analysis (Additional file 7: Figure S6), and both were calculated to have correct protein expression (Fig. 4a) at levels of approximately 1 mg/mL, calculated by the same method as for pCSF1-Fc. Purification was identical to that of pCSF1-Fc, including recovery efficiency (Fig. 4b). Since hCSF1 has previously been shown to interact with pig bone marrow [34], the bone marrow differentiation assay was repeated to test activity of purified hCSF1-Fc, and protein from both lines was shown to promote survival and differentiation at an equivalent level to each other and pCSF1-Fc control, even after vacuum drying and reconstitution (Fig. 4c).

These results indicate that both interferon α2a and CSF1-Fc are efficiently synthesized as a component of egg white, readily recoverable using common chromatography methods, and biologically active in vitro and in vivo at levels equal to control cytokines produced in different systems.

## Discussion

Lentiviruses are efficient vectors for the generation of transgenic chickens with stable germline transmission and consistent, oviduct-specific expression of correctly folded and functional protein [17]. Here we have demonstrated an improved promoter design that generated levels of expression compatible with cost-effective scale-up. There were no negative effects on the health of the hens despite the substantial increase in expression of exogenous protein. The method can be applied to untagged and Fc-tagged proteins, and common chromatographic techniques can be applied to purify the proteins from egg white. Initial characterization of egg-derived proteins showed high levels of purity and activity as good as or better than the equivalent proteins produced in traditional cell culture or *E. coli* systems. Contamination by egg proteins was below levels detectable by mass spectrometry and in vivo testing showed no immunogenic reaction in mice. It is likely that similarly-structured proteins, including the many therapeutic Fc fusion proteins currently under clinical evaluation [23, 24]. will be amenable to production in the egg white of transgenic chickens and purification as we have described at commercially relevant scale.

The infrastructure and upstream costs of a transgenic chicken bioreactor production system are predicated to be substantially lower than those for mammalian cell culture, perhaps by as much as two orders of magnitude, due largely to the comparably lower cost of chicken facilities and lack of requirement for GMP conditions until egg crack. Initial analysis provided by the Centre for Process Innovation National Biologics Manufacturing Centre (Darlington, UK) indicates that downstream processing costs should be equivalent to current methods, leading to an overall lower manufacturing cost. Relative to mammalian transgenic animal bioreactors, a flock of bioreactor chickens is faster and cheaper to produce, and egg white is a simpler mixture of proteins with a significantly lower lipid content than milk. In addition, the favorable glycosylation patterns and oxidative environment of the chicken system make it appealing for production of proteins with specific requirements for these characteristics that are currently difficult to recapitulate in cell culture.

The lentivirus system was the best available at the time of generation of these chicken lines, but since then, significant advances have taken place in chicken primordial germ cell culture and genome editing. Combining these techniques means precise control over the insertion site of the transgene and uniformity of the resulting chicken population. It also means substantially increased efficiency, reduced need for genotype screening, no biosafety concerns around lentiviral particles, and overall lower upstream line generation costs, making the transgenic chicken bioreactor an even more appealing option.

Human interferons were amongst the first patented biologicals. The extension of many of the therapeutic applications of human interferon α2a (RoferonA) and other isoforms to companion animal veterinary medicine have been reviewed recently [36]. Interferon also has increased bioavailability as a Fc fusion protein [25], and this form would be even more readily produced in a chicken bioreactor using the purification approach described here. Production in chickens may bring the cost down to levels that would support wider applications, and extension to containment of viral diseases in valuable livestock. For the CSF1-Fc fusion protein, a dose of 1 mg/kg twice a week was required to produce a maximal elevation of blood monocyte and tissue macrophage numbers when tested in pigs [31]. Presuming similar efficacy in humans, a single dose could be obtained from 2 to 3 eggs. One of the therapeutic applications of CSF1-Fc already validated in mouse models is in the treatment of bone fractures [37], an application of relevance to both human and companion animal medicine. Even if production of proteins such as CSF1-Fc in chickens faces hurdles in translation to the human clinic, the bioreactor will enable cost-effective production of the amounts required to test clinical applications in informative large animal models.

## Conclusions

We have demonstrated that a human cytokine, interferon α2a, can be recovered at a level of 15 mg/100 ml egg white (the approximate volume from 3 eggs) with biological activity estimated as 1 × 10^9^ IU/mg. In addition, we have generated two transgenic lines in which porcine and human CSF1 have been synthesized as FC fusion proteins. Very high recovery (80–90%) from ~ 100 mg/100 ml of highly pure proteins with biological activity comparable to equivalent proteins produced in cell culture was demonstrated. We have validated the transgenic chicken system for the cost-effective production of pure, high quality, biologically active proteins for therapeutics and other applications.

## Methods

### Construction of transgenes

The human interferon α2a coding sequence was codon-optimized for chicken and synthesized with a Kozak consensus sequence, start codon and chicken lysozyme signal peptide at the 5′ end. The synthetic gene was ligated into a replication defective vector derived from equine infectious anemia virus (EIAV) containing a 4.4 kb modified form of the EREOVA promoter and regulatory elements (EREOVA2) previously shown to drive protein expression restricted to the hen oviduct [17]. The original EREOVA promoter had 0.9 kb deleted between the estrogen-responsive enhancer element (ERE) and the steroid-dependent regulatory element (SDRE) that was preserved in EREOVA2. The new promoter was found to enhance protein expression in egg white at least 10-fold compared to EREOVA. The synthesized, codon-optimized gene for pCSF1-Fc (Entelechon, Germany) consisted of a Kozak sequence, start codon, lysozyme signal peptide and 154 amino acid active form of porcine CSF1 joined to the hinge-CH3 region of porcine IgG1a as previously described (13). This transgene was cloned using designed restriction sites AatII and NheI into a modified pLenti6/DEST Gateway vector, with the V5 tag removed and downstream oPRE added. This vector was derived from human immunodeficiency virus (HIV) and contained the EREOVA2 promoter.

### Production of transgenic birds

The birds were derived from a flock of Novagen Brown commercial egg layer strain. Lentiviral vectors were packaged and injected into embryos from new-laid eggs to generate G_0_ transgenic birds [17]. Cockerels estimated by PCR analysis to have vector sequence present in the germ line at frequency of > 1% were bred with wild-type hens to generate G_1_ birds, which were screened by PCR and Southern transfer analysis to confirm that the complete gene was present as a single copy. Egg white from G_1_ hens or female G_2_ offspring of G_1_ cockerels was tested for presence of transgene-derived protein. Lines were established from validated G_1_/G_2_ birds for each transgene.

### Protein expression analysis

Protein expression was demonstrated by SDS-PAGE and western blot. Samples were prepared by addition of 4x NuPAGE LDS sample buffer, and 10x sample reducing agent for gels that were run under reducing conditions and heated at 70 °C for 10 min before gel electrophoresis using Novex NuPAGE 12% Bis-Tris/MOPS gels (Life Technologies). Proteins were transferred onto nitrocellulose membranes by use of an XCell II Blot Module (Life Technologies). Transfer was verified by Ponceau S staining and membranes were blocked overnight at 4 °C. For westerns imaged using the LI-COR scanner, blocking was performed with Odyssey Blocking Buffer, while for westerns visualized using HRP, blocking was performed with 5% (*w*/*v*) powdered milk in TBS. Interferon α2a was detected with monoclonal anti-interferon α (Sigma, UK, catalogue number SAB1409236) at 1:1000 dilution and sheep anti-mouse IgG conjugated HRP (GE Healthcare NXA931-1ML) or LI-COR IRDye 680RD Donkey anti-Rabbit IgG (H + L) at 1:20,000 dilution and imaged with a LI-COR Odyssey Imager according to manufacturer’s instructions. Concentration was estimated with a commercial ELISA kit according to manufacturer’s instructions (PBL Biomedical Laboratories 41,100). Due to a lack of antibody against porcine CSF1, CSF1-Fc was immunoblotted with anti-porcine Fc conjugated to HRP (Bethyl Laboratories A100-104P) at a 1:10,000 dilution in 3% (w/v) powdered milk in TBS 0.1% (*v*/v) Tween-20. Interferon concentration was determined by quantitative Western blot using LI-COR Image Studio software.

### Egg white activity analysis

Whole egg white from transgenic hens’ eggs was tested for activity prior to purification. For interferon, the ability to stimulate interferon-stimulated response element (ISRE)-driven transcription of luciferase was used to determine initial whole egg white activity. The pGL4.45[luc2P/ISRE/Hygro] vector (Promega) was transfected in HEK293T cells seeded in white-walled 96-well plates at 1 × 10^4^ cells/well using FuGENE HD (Promega) according to manufacturer’s instructions. 24 h post-transfection, the medium was replaced with antibiotic-free medium and either egg white from transgenic hens or wild type egg white with control interferon protein (Sigma) added at an estimated final concentration of 10 international units (IU), 10^2^ IU and 10^4^ IU, with diluted wild type egg white used as a negative control. After a further 24 h, the One Glo Luciferase assay kit (Promega) was used to measure luminescence according to manufacturer’s instructions. For pCSF1-Fc, a cell survival assay based upon the ability of viable cells to reduce 3-(4,5-dimethylthiazol-2-yl)-2,5-diphenyltetrazolium bromide (MTT) was performed as previously described [30]. Briefly, stable Ba/F3 cells expressing porcine CSF1 receptor were grown in complete RPMI (10% (*v*/v) fetal calf serum, penicillin, streptomycin, and GlutaMAX) and supplemented with 5% (v/v) X63 cell supernatant from X63 Ag8–653 myeloma cells carrying an expression vector for IL-3 (22). Cells were seeded in 96-well plates at a density of 2 × 10^4^ cells/well in triplicate and treated with serial dilutions of egg or CHO cell-produced pCSF1-Fc instead of IL-3, with negative controls containing complete RPMI with no X63 or pCSF1-Fc. Plates were incubated at 37 °C, 5% (*v*/v) CO_2_ for 48 h and MTT (Sigma) at a final concentration of 0.5 mg/mL was added for a further 3-h incubation, followed by solubilization overnight with acidified isopropanol. Absorbance was measured at 570 nm and results normalized to the highest dose positive control.

### Protein purification and analysis

For pCSF1-Fc and hCSF1-Fc purification, egg white was diluted 1:4 in 50 mM phosphate buffer pH 3, adjusted to pH 4, and stirred at room temperature for 10 min to precipitate ovomucin. The diluted egg white was centrifuged at 25,000 g for 45 min at 10 °C, the supernatant adjusted to pH 7 and centrifuged again at 25,000 g for 45 min at 10 °C to remove any further precipitates. The clarified egg white was diluted to a final volume of 10 times the original egg white volume and pCSF1-Fc was bound to a 5 mL HiTrap MabSelect SuRe column (GE Healthcare) pre-equilibrated with 50 mM phosphate buffer, pH 7, (PB) at 5 mL/min by use of an Äkta Prime Plus liquid chromatography system (GE Healthcare). The column was washed with 10 column volumes of PB and the protein was eluted with 3–5 column volumes of 100 mM citrate buffer (CB) pH 3, or until the UV peak had returned to baseline, into tubes containing 1 M Tris pH 9 at 30% of the final fraction volume. The column was regenerated with 3 column volumes of CB and 5 column volumes of PB, cleaned in place with 5 column volumes of 0.5 M NaOH, pH adjusted with 5 column volumes of PB and finally cleaned with 10 column volumes of 20% (*v*/v) ethanol for storage. The fractions containing eluted protein, as determined by SDS-PAGE, were pooled and concentrated to < 13 mL in an Amicon stirred cell with 10,000 Da ultrafiltration regenerated cellulose membrane and injected onto a Superdex 200 26/600 column (GE Healthcare) equilibrated with PBS at 2.5 mL/min by use of the Äkta Prime Plus. Fractions were collected manually to ensure that only the main portion of the correct peak was isolated. After use the column was cleaned with 1–2 column volumes of 20% (v/v) ethanol and stored.

For Interferon α2a, egg white was diluted 1:4 in 20 mM phosphate buffer pH 3, adjusted to pH 4, and stirred at room temperature for 10 min to precipitate ovomucin. The diluted egg white was then centrifuged at 25,000 g for 45 min at 10 °C, the supernatant adjusted to pH 7 and centrifuged again at 25,000 g for 35 min at 10 °C to remove any further precipitates. The clarified egg white was diluted to a final volume of 10 times the original egg white volume and interferon α2a was bound onto a 5 mL HiTrap Blue column (GE Healthcare) equilibrated with 20 mM PB pH 7 at 5 mL/min by use of an Äkta Prime Plus liquid chromatography system (GE Healthcare). The column was washed with 10 column volumes of PB and protein eluted with 20 mM PB containing 1 M NaCl. The fractions containing interferon α2a, as determined by SDS-PAGE, were pooled, diluted to 10 times the pooled volume in ddH_2_O to prevent salt-induced association of interferon and ovalbumin, concentrated to < 13 mL in an Amicon stirred cell with 10,000 Da ultrafiltration regenerated cellulose membrane and injected onto a Superdex 200 26/600 column (GE Healthcare) equilibrated with PBS at 2.5 mL/min by use of the Äkta Prime Plus. After use the column was cleaned with 1–2 column volumes of 20% (*v*/v) ethanol and stored. Interferon-containing fractions, as determined by SDS-PAGE, were concentrated by Amicon filtration. Protein concentration was determined by UV_280_. Identity was confirmed by SDS-PAGE and mass spectrometric analyzes (Roslin Proteomics and Metabolomics Facility) and quality was assessed by reversed phase chromatography and SEC-multi-angle light scattering at the Edinburgh Protein Production Facility (EPPF), Kings Buildings, Edinburgh.

### Analysis of purified Interferon α2a activity

A dose-inhibition assay was performed to determine interferon activity compared to a commercially available control (Sigma SRP4594). HEK 293 T cells were used to produce influenza A/PR/8/34 (PR8) virus as described previously (17). A549 cells were pre-treated with varying concentrations of interferon for 24 h, then infected at low multiplicity (MOI 0.001). After 1 h of adsorption at 37 °C, virus-containing medium was replaced with virus growth medium (DMEM, 1 μg/mL TPCK-treated trypsin, 0.14% (*w*/*v*) BSA). After 48 h, virus was harvested and titer determined by plaque assay on MDCK cells with an Avicel overlay (virus growth medium supplemented with 1.2% (v/v) Avicel) followed by toluidine blue staining (17, 23). The virus titer was normalized to a no-interferon control to determine dose-inhibition.

### Analysis of purified pCSF1-Fc activity

In vitro activity of purified egg pCSF1-Fc was tested by the MTT assay in Ba/F3 cells as described for whole egg white, and in pig bone marrow cells as previously described [30]. Briefly, pig bone marrow cells were derived from pigs culled by captive bolt at Dryden farm as previously described [31, 38]. Cells were plated at 5 × 10^4^ cells/well in a 96-well plate directly from frozen stocks and grown in complete RPMI supplemented with either CHO-derived or egg purified pCSF1-Fc at 10 ng/mL, 100 ng/mL or 1000 ng/mL. After 7 days, the culture medium was replaced with 50 μl of 1 mg/ml MTT solution and incubated for 1 h at 37 °C. The MTT solution was removed and tetrazolium salt solubilized with 100 μl of solubilization agent (0.1 M HCL, 10% Triton X-100 and isopropanol) followed by incubation at 37 °C with 5% CO2 for 10 min. Plates were read at 570 nm.

In vivo activity of pCSF1-Fc was tested in mice as previously described [30]. Approval was obtained from The Roslin Institute’s and The University of Edinburgh’s Protocols and Ethics Committees. The experiments were carried out under the authority of a UK Home Office Project License under the regulations of the Animals (Scientific Procedures) Act 1986. Twelve male C57BL/6 mice aged 6–8 weeks were purchased from Charles River Laboratories (UK) and injected subcutaneously with either PBS, 1 mg/kg pCSF1-Fc from CHO cells or 1 mg/kg pCSF1-Fc from eggs (*n* = 4 per group) for 4 days and sacrificed on day 5 by rising levels of carbon dioxide. Liver and spleen were removed, weighed and fixed in 10% neutral buffered formalin for histological analysis. Blood was collected by cardiac puncture and transferred into EDTA-coated tubes, then prepared for flow cytometry using the Uti-Lyse kit (Dako, Ely, UK) and stained with PE anti-mouse F4/80 (Biolegend, CA, USA, catalogue number 122616, 1:200) and APC anti-mouse Mac1/CD11b (Abcam, Cambridge, UK, catalogue number ab25482, 1:2000) to identify the macrophage population. Results were analyzed with FlowJo software.

### Analysis of purified hCSF1-Fc activity

Activity of purified egg hCSF1-Fc was tested by the bone marrow MTT assay as described for pCSF1-Fc. To test whether the protein could withstand drying for long-term storage and easier shipping, aliquots of protein were brought to dryness using a Savant SPD2010 Speedvac (Thermo) at a constant vacuum pressure of 5.1 Torr over 1.5-h without heating. The protein was reconstituted in the same volume of ddH_2_O and used as described for pCSF1-Fc in the bone marrow differentiation assay.

### Statistical analysis

Analysis was performed with GraphPad Prism Version 6.04 and statistical significance was assessed using Student’s two-tailed t-test. Resulting values were considered statistically significant at *p* < 0.05.

## Additional files

Additional file 1: **Figure S1.** EREOVA2 sequence. DNA sequence of EREOVA2 promoter. (DOCX 16 kb)

Additional file 2: **Figure S2.** Interferon α2A mono-dispersity, size and purity analysis. A) Size-exclusion chromatography multi-angled light scattering (SEC-MALS) was used to determine the molecular and mono-dispersity of Interferon α2A in solution. Interferon α2A eluted from a Superdex-200 Increase 10/300 GL size exclusion column as a single peak with slight trailing edge (≥ 96% purity; As > 2) with apparent molecular mass of ~ 33.2 ± 2 kDa and an Rs of 2.43 ± 0.2 nm (mean ± SD, *n* = 3). The molecular mass average across the elution profile is 18.7 kDa with average mono-dispersity (Mw/Mn = 1.038) and no significant aggregation. B) Elution profile of Interferon α2A from an RPC 5 4.6/150 ST column run. Chromatogram shows single sharp peak, with ≥98% purity. (DOCX 486 kb)

Additional file 3: **Figure S3.** Confirmation of pCSF1-Fc transgene and copy number in G1 birds by Southern transfer analysis, Southern transfer analysis of genomic DNA from individual G1 birds. A) Samples from 6 birds positive by PCR for HIV sequence were digested with BamHI (located at the 5′ end of the promoter) and AvrII (located at the 3′ end of the oPRE) to generate a 6.6 kb fragment spanning most of the ovalbumin promoter and the pCSF1-Fc coding sequence. Any smaller bands were considered to be truncated versions of the transgene.B) Samples from the three birds containing intact transgenes were digested with BamHI to detect insertion events, with each event expected to show a distinct band. (DOCX 7376 kb)

Additional file 4: **Figure S4.** Comparison of expression between two generations and different individuals of pCSF1-Fc birds. 5 μL egg white diluted 1:50 from a second generation pen (lane 1) and three third generation pens (lanes 3–5) of pCSF1-Fc, compared by non-reducing western blot against each other and 500 ng of purified pCSF1-Fc from second generation egg white. (DOCX 267 kb)

Additional file 5: **Figure S5.** pCSF1-Fc mono-dispersity, size and purity analysis**.** A) Size-exclusion chromatography multi-angled light scattering (SEC-MALS) was used to determine the molecular and mono-dispersity of pCSF1-Fc in solution. pCSF1-Fc eluted as a single peak (≥ 97% purity; As = 0.91) from a Superdex-200 Increase 10/300 GL size exclusion column with apparent molecular mass of ~ 110 ± 5 kDa and an Rs of 4.03 ± 0.4 nm (mean ± SD, n = 3). B) Elution profile of pCSF1-Fc from an RPC 5 4.6/150 ST column run. Chromatogram shows single sharp peak, with ≥98% purity. (DOCX 188 kb)

Additional file 6: **Table S1.** pCSF1-Fc purity assessed by LC-MS/MS analysis. The purified protein sample was digested in-solution using trypsin and analyzed on a micrOTOF-QII (Bruker) mass spectrometer. The spectral data was searched against Uniprot porcine protein sequence database to confirm the identity of pCSF1-Fc and against Uniprot chicken database to check the purity from chicken egg proteins using Mascot server. (A composite file of results from separate Mascot searches of LC-MS/MS data.) (DOCX 332 kb)

Additional file 7: **Figure S6.** Confirmation of hCSF1-Fc transgene and copy number in G1 birds by Southern transfer analysis. Southern transfer analysis of genomic DNA from individual G1 birds. A) Samples from 7 birds positive by PCR for HIV sequence were digested with BamHI (located at the 5′ end of the promoter) and AgeI (located at the 3′ end of the oPRE) to generate a 6.3 kb fragment spanning most of the ovalbumin promoter and the hCSF1-Fc coding sequence. B) Samples from the seven birds were digested with BamHI to detect insertion events, with each event expected to show a distinct band. (DOCX 15849 kb)

## Abbreviations

CSF1: Colony stimulating factor 1
EIAV: Equine infectious anemia virus
EPO: Erythropoietin
ERE: Estrogen-responsive enhancer element
G-CSF: Granulocyte colony stimulating factor
GM-CSF: Granulocyte-macrophage colony stimulating factor
GMP: Good manufacturing process
ISRE: Interferon-stimulated response element
IU: International units
LSP: Lysozyme signal peptide
LTR: Long terminal repeats
oPRE: Optimized woodchuck hepatitis virus posttranscriptional regulatory element
SDRE: Steroid-dependent regulatory element
SEC: Size exclusion chromatography
