# Supplementary material for: A chicken bioreactor for efficient production of functional cytokines
Source: BMC Biotechnol. 2018 Dec 29;18:82. doi: 10.1186/s12896-018-0495-1 (PMC6311007; doi:10.1186/s12896-018-0495-1)
Supplement: Supplementary file 1 — Figure S1. EREOVA2 sequence. DNA sequence of EREOVA2 promoter. (DOCX 16 kb) [file 12896_2018_495_MOESM1_ESM.docx]

Additional file 1

tctcttcagaatggcacagcaccgctgcagaaaaatgccaggtggactatgaactcacatccaaaggagcttgacctgatacctgattttcttcaaacaggggaaacaacacaatcccacaaaatagctcagagagaaaccatcactgatggctacagcaccaaggtatgcaatggcaatccattcgacattcatctgtgacctgagcaaaatgatttatctctccatgaatggttgcttctttccctcatgaaaaggcaatttccacactcacaatatgcaacaaagacaaacagagaacaattaatgtgctccttcctaatgtcaaaattgtagtggcaaagaggagaacaaaatctcaagttctgagtaggttttagtgattggataagaggctttgacctgtgagctcacctggacttcatatccttttggataaaaagtgcttttataactttcaggtctccgagtctttattcatgagactgttggtttagggacagacccacaatgaaatgcctggcataggaaagggcagcagagccttagctgaccttttcttgggacaagcattgtcaaacaatgtgtgacaaaactatttgtactgctttgcacagctgtgctgggcagggcaatccattgccacctatcccaggtaaccttccaactgcaagaagattgttgcttactctctctagacccccaagtcaaaccaactatgcaggtatgctgacaacgctatgatgacagcctgttctgatcaagatctcatttgttcatggacaatttttgttgcttgcagctggtcttccattgggaaagagtgtagtatatccttctcatctgacagaaaagcagaaattctcatgctccacacttaatctacattgttttaaaccaccagctacttcttggagaggaaaaatggcttttataagactcacaaaacaaagctctgcaagtcaaatgcatacaaaactgttctgtaggtctggaatcaggacactatgtggaagtcaaatagagaagctttaaaaaaacctttgggatcattctcatcttatatttgcagcacgatactatgacagtgataactgacataactgcatcaatttccttgatattttatttgtcttaaagtacaagacatagagatggacgtaaagatggacatatgactcaggtctggacaggtccgtggtccatgtatgataaaagagatgaagggaaggagaatggagactgtctaagaagggcttcagggacgttctgaaggcagatttgactgaatcagatgtactgtccaagtctcatatgtagcaatggaagactgatattggagaaatataaagaaatggctgtgaactcaaagtgaccctgaacagaaaagggatatggagttaaaataatggcacagaactgaggtttatatgatataccatgggctgcagagggtcagagtgctccaccatgggcctctcttgggctgcagggaacttctgttctacacctggaacacctcctgccctcctccgcactgacctcagtgtcatcagggctgtttctctcacattttctcactcacctctcccaactaccattgtacagcagttgttcttacatcttgctcctcctgaggtgcatctagcatttaagtcctcagacttggcaaggagaatgtagatttctacagtatatatgttttcacaaaaggaaggagagaaacaaaagaaaatggcactgactaaacttcagctagtggtataggaaagtaattctgcttaacagagattgcagtgatctctatgtatgtcctgaagaattatgttgtacttttttcccccatttttaaatcaaacagtgctttacagaggtcagaatggtttctttactgtttgtcaattctattatttcaatacagaacaatagcttctataactgaaatatatttgctattgtatattatgattgtccctcgaaccatgaacactcctccagctgaatttcacaattcctctgtcatctgccaggccattaagttattcatggaagatctttgaggaacactgcaagttcatatcataaacacatttgaaattgagtattgttttgcattgtatggagctatgttttgctgtatcctcagaaaaaaagtttgttataaagcattcacacccataaaaagatagatttaaatattccagctataggaaagaaagtgcgtctgctcttcactctagtctcagttggctccttcacatgcatgcttctttatttctcctattttgtcaagaaaataataggtcacgtcttgttctcacttatgtcctgcctagcatggctcagatgcacgttgtagatacaagaaggatcaaatgaaacagacttctggtctgttactacaaccatagtaataagcacactaactaataattgctaattatgttttccatctctaaggttcccacatttttctgttttcttaaagatcccattatctggttgtaactgaagctcaatggaacatgagcaatatttcccagtcttctctcccatccaacagtcctgatggattagcagaacaggcagaaaacacattgttacccagaattaaaaactaatatttgctctccattcaatccaaaatggacctattgaaactaaaatctaacccaatcccattaaatgatttctatggcgtcaaaggtcaaacttctgaagggaacctgtgggtgggtcacaattcaggctatatattccccagggctcagccagtgtctgtacatacagctagaaagctgtattgcctttagcagtcaagctcgaaaggtaagcaactctctggaattaccttctctctatattagctcttacttgcacctaaactttaaaaaattaacaattattgtgctatgtgttgtatctttaagggtgaagtacctgcgtgataccccctataaaaacttctcacctgtgtatgcattctgcactattttattatgtgtaaaagctttgtgtttgttttcaggaggcttattctttgtgcttaaaatatgtttttaatttcagaacatcttatcctgtcgttcactatctgatatgctttgcagtttgcttgattaacttctagccctacagagtgcacagagagcaaaatcatggtgttcagtgaattctggggagttattttaatgtgaaaattctctagaagtttaattcctgcaaagtgcagctgctgatcactacacaagataaaaatgtggggggtgcataaacgtatattcttacaataatagatacatgtgaacttatatacagaaaagaaaatgagaaaaatgtgtgtgtgtatactcacacacgtggtcagtaaaaacttttgaggggtttaatacagaaaatccaatcctgaggccccagcactcagtacgcatataaagggctgggctctgaaggacttctgactttcacagattatataaatctcaggaaagcaactagattcatgctggctccaaaagctgtgctttatataagcacactggctatacaatagttgtacagttcagctctttataatagaaacagacagaacaagtataaatcttctattggtctatgtcatgaacaagaattcattcagtggctctgttttatagtaaacattgctattttatcatgtctgcatttctcttctgtctgaatgtcaccactaaaatttaactccacagaaagtttatactacagtacacatgcatatctttgagcaaagcaaaccatacctgaaagtgcaatagagcagaatatgaattacatgcgtgtctttctcctagactacatgaccccatataaattacattacttatctattctgccatcaccaaaacaaaggtaaaaatacttttgaagatctactcatagcaagtagtgtgcaacaaacagatatttctctacatttatttttagggaataaaaataagaaataaaatagtcagcaagcctctgctttctcatatatctgtccaaacctaaagtttactgaaatttgctctttgaatttccagttttgcaagcctatcagattgtgttttaatcagaggtactgaaaagtatcaatgaattctagctttcactgaacaaaaatatgtagaggcaactggcttctgggacagtttgctacccaaaagacaactgaatgcaaatacataaatagatttatgaatatggttttgaacatgcacatgagaggtggatatagcaacagacacattaccacagaattactttaaaactacttgttaacatttaattgcctaaaaactgctcgtaatttactgttgtagcctaccatagagtaccctgcatggtactatgtacagcattccatccttacattttcactgttctgctgtttgctctagacaactcagagttcacc
