# Supplementary figures and images for: A chicken bioreactor for efficient production of functional cytokines
Source: BMC Biotechnol. 2018 Dec 29;18:82. doi: 10.1186/s12896-018-0495-1 (PMC6311007; doi:10.1186/s12896-018-0495-1)

Additional file 2


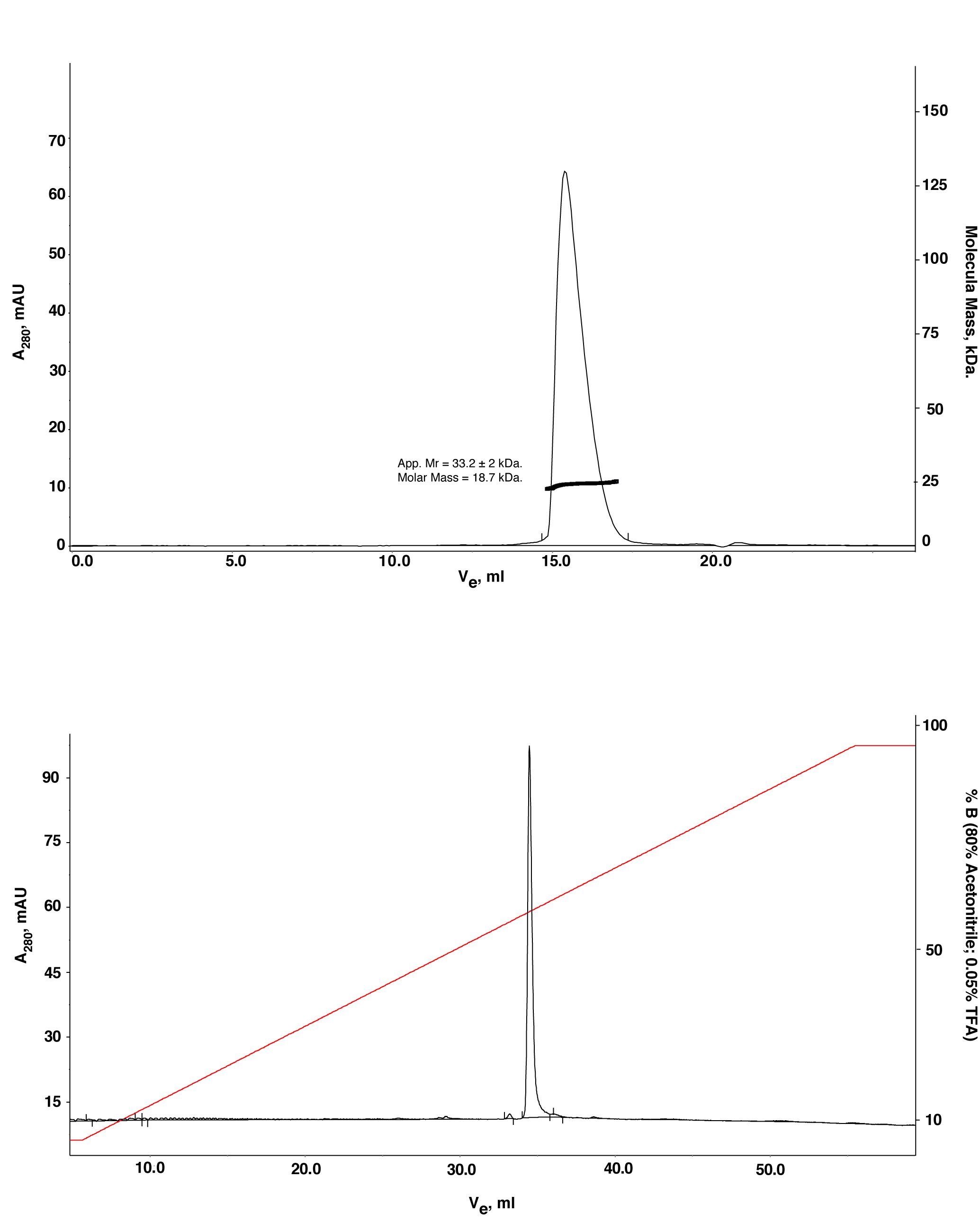


**a**

**b**

Supplement: Supplementary file 2 — Figure S2. Interferon α2A mono-dispersity, size and purity analysis. A) Size-exclusion chromatography multi-angled light scattering (SEC-MALS) was used to determine the molecular and mono-dispersity of Interferon α2A in solution. Interferon α2A eluted from a Superdex-200 Increase 10/300 GL size exclusion column as a single peak with slight trailing edge (≥ 96% purity; As > 2) with apparent molecular mass of ~ 33.2 ± 2 kDa and an Rs of 2.43 ± 0.2 nm (mean ± SD, n = 3). The molecular mass average across the elution profile is 18.7 kDa with average mono-dispersity (Mw/Mn = 1.038) and no significant aggregation. B) Elution profile of Interferon α2A from an RPC 5 4.6/150 ST column run. Chromatogram shows single sharp peak, with ≥98% purity. (DOCX 486 kb) [file 12896_2018_495_MOESM2_ESM.docx]

Additional file 3

**a**

**b**

Lane

Lane


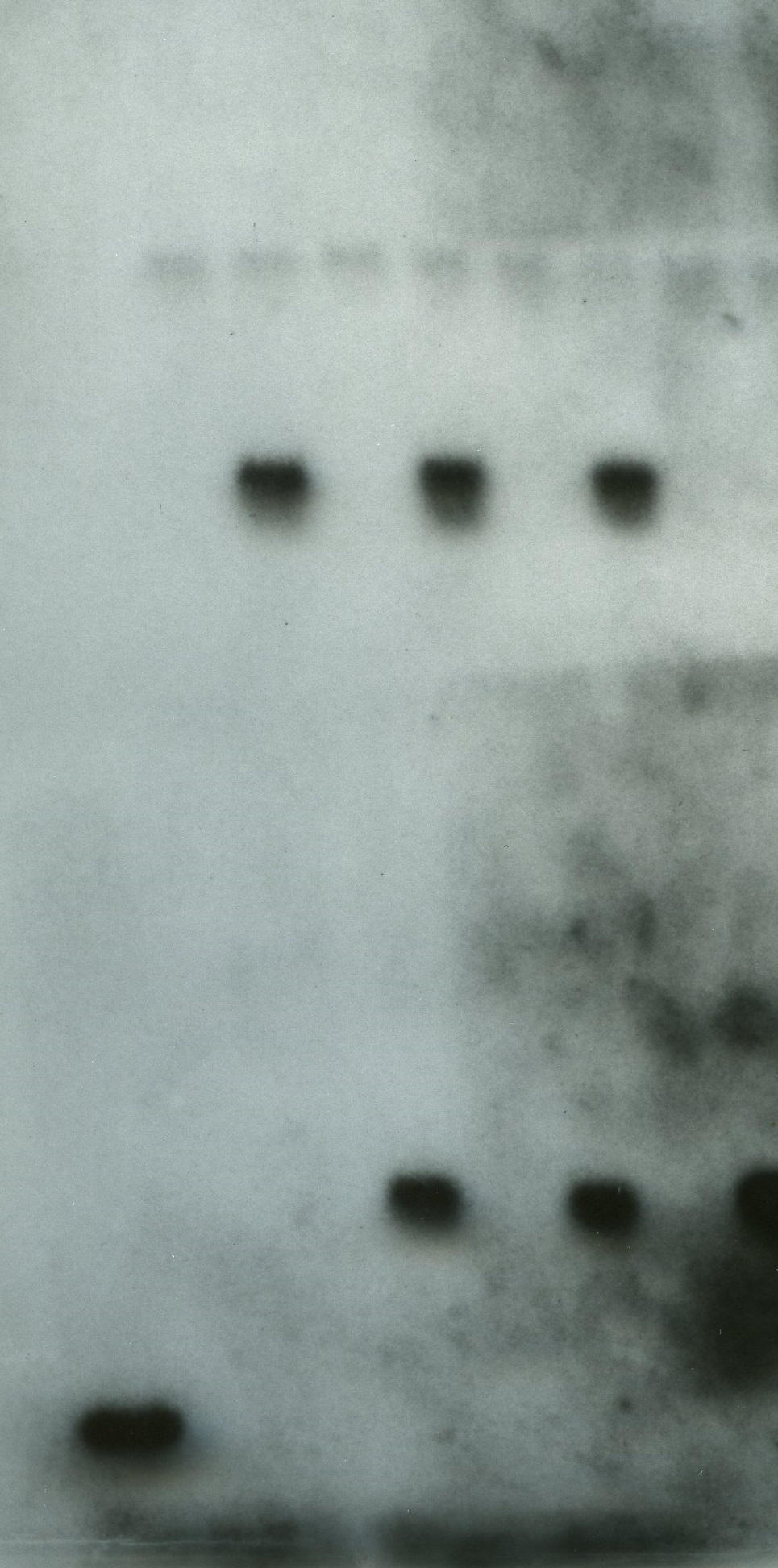


5kb

1

2

3

4

5

6

1kb

ladder

BamHI and AvrII


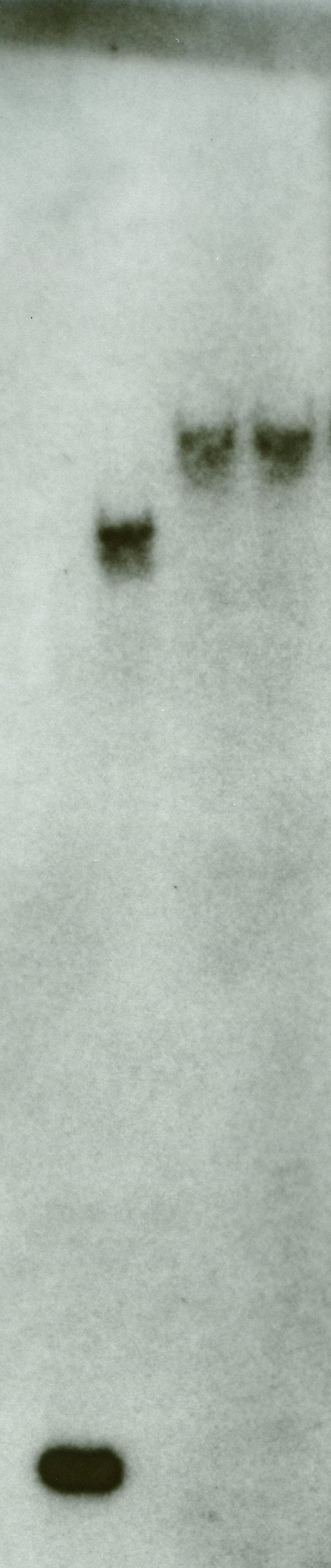


1

2

3

1kb

ladder

BamHI

Supplement: Supplementary file 3 — Figure S3. Confirmation of pCSF1-Fc transgene and copy number in G1 birds by Southern transfer analysis, Southern transfer analysis of genomic DNA from individual G1 birds. A) Samples from 6 birds positive by PCR for HIV sequence were digested with BamHI (located at the 5′ end of the promoter) and AvrII (located at the 3′ end of the oPRE) to generate a 6.6 kb fragment spanning most of the ovalbumin promoter and the pCSF1-Fc coding sequence. Any smaller bands were considered to be truncated versions of the transgene.B) Samples from the three birds containing intact transgenes were digested with BamHI to detect insertion events, with each event expected to show a distinct band. (DOCX 7376 kb) [file 12896_2018_495_MOESM3_ESM.docx]

Additional file 4


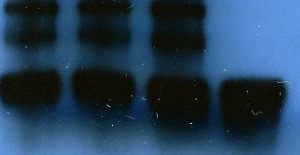

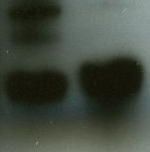


191

97

1:50 5μL

1:50 5μL

Pure egg

pCSF1-Fc

500ng

Pure egg

pCSF1-Fc

500ng

1

2

3

4

5

6

kDa

Supplement: Supplementary file 4 — Figure S4. Comparison of expression between two generations and different individuals of pCSF1-Fc birds. 5 μL egg white diluted 1:50 from a second generation pen (lane 1) and three third generation pens (lanes 3–5) of pCSF1-Fc, compared by non-reducing western blot against each other and 500 ng of purified pCSF1-Fc from second generation egg white. (DOCX 267 kb) [file 12896_2018_495_MOESM4_ESM.docx]

Additional file 5


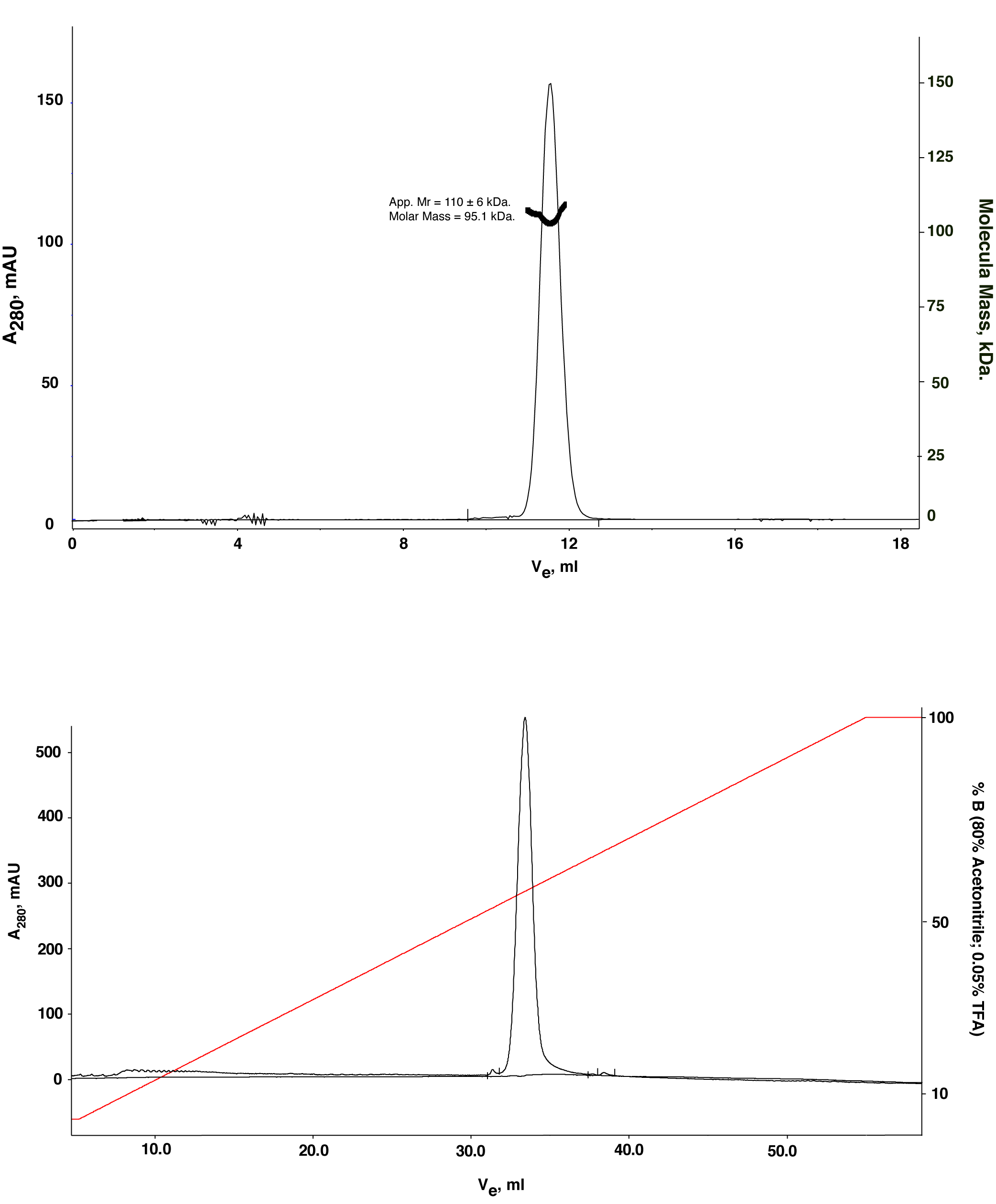


**a**

**b**

Supplement: Supplementary file 5 — Figure S5. pCSF1-Fc mono-dispersity, size and purity analysis. A) Size-exclusion chromatography multi-angled light scattering (SEC-MALS) was used to determine the molecular and mono-dispersity of pCSF1-Fc in solution. pCSF1-Fc eluted as a single peak (≥ 97% purity; As = 0.91) from a Superdex-200 Increase 10/300 GL size exclusion column with apparent molecular mass of ~ 110 ± 5 kDa and an Rs of 4.03 ± 0.4 nm (mean ± SD, n = 3). B) Elution profile of pCSF1-Fc from an RPC 5 4.6/150 ST column run. Chromatogram shows single sharp peak, with ≥98% purity. (DOCX 188 kb) [file 12896_2018_495_MOESM5_ESM.docx]

Additional file 6


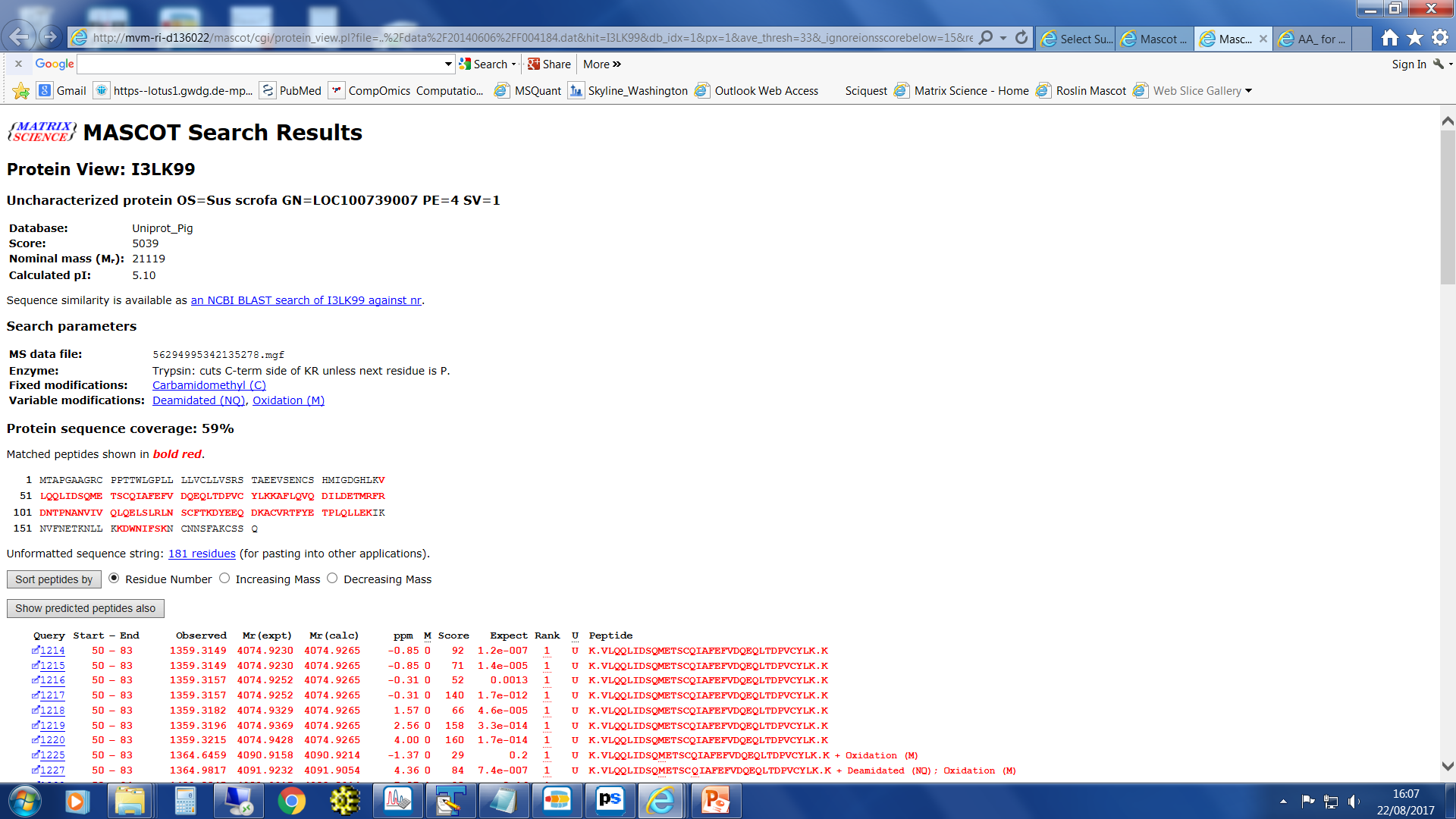

Supplement: Supplementary file 6 — Table S1. pCSF1-Fc purity assessed by LC-MS/MS analysis. The purified protein sample was digested in-solution using trypsin and analyzed on a micrOTOF-QII (Bruker) mass spectrometer. The spectral data was searched against Uniprot porcine protein sequence database to confirm the identity of pCSF1-Fc and against Uniprot chicken database to check the purity from chicken egg proteins using Mascot server. (A composite file of results from separate Mascot searches of LC-MS/MS data.) (DOCX 332 kb) [file 12896_2018_495_MOESM6_ESM.docx]

Additional file 7


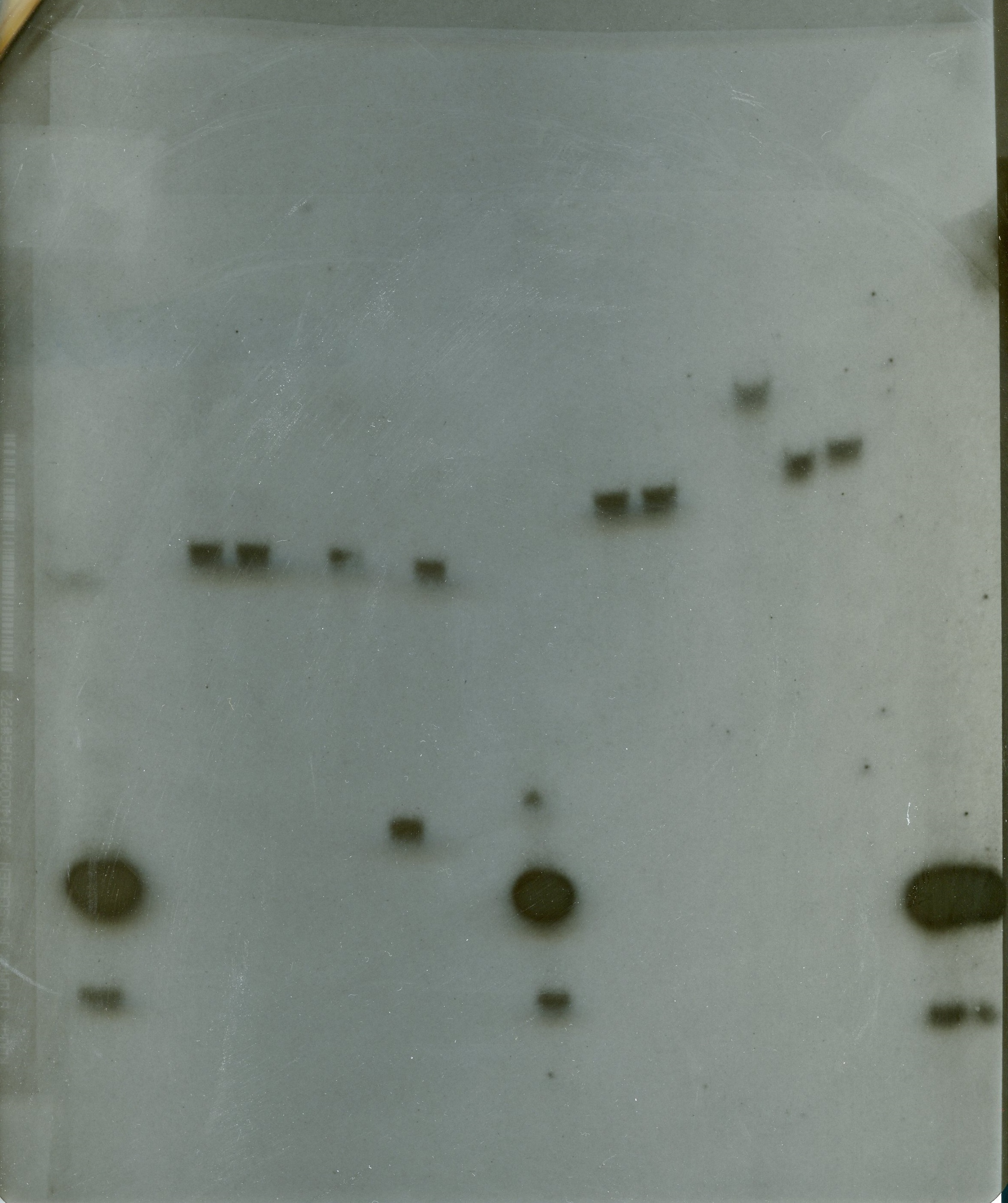


Lane

6kb

1

2

3

4

5

7

1kb

ladder

BamHI and AgeI

BamHI

6


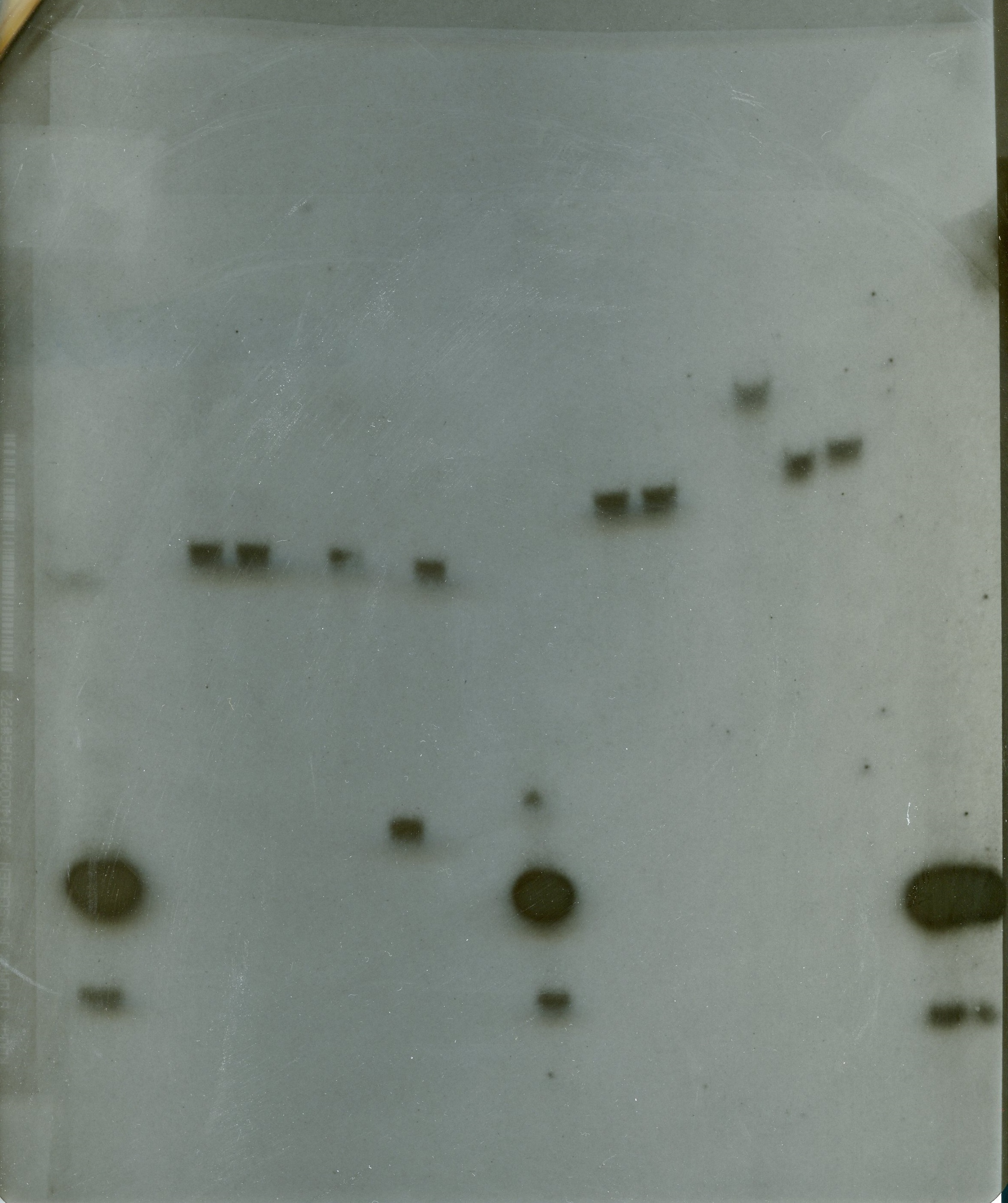


1

2

3

4

5

7

1kb

ladder

6

Supplement: Supplementary file 7 — Figure S6. Confirmation of hCSF1-Fc transgene and copy number in G1 birds by Southern transfer analysis. Southern transfer analysis of genomic DNA from individual G1 birds. A) Samples from 7 birds positive by PCR for HIV sequence were digested with BamHI (located at the 5′ end of the promoter) and AgeI (located at the 3′ end of the oPRE) to generate a 6.3 kb fragment spanning most of the ovalbumin promoter and the hCSF1-Fc coding sequence. B) Samples from the seven birds were digested with BamHI to detect insertion events, with each event expected to show a distinct band. (DOCX 15849 kb) [file 12896_2018_495_MOESM7_ESM.docx]
